# Blunt trauma induced splenic blushes are not created equal

**DOI:** 10.1186/1749-7922-7-8

**Published:** 2012-03-30

**Authors:** Clay Cothren Burlew, Lucy Z Kornblith, Ernest E Moore, Jeffrey L Johnson, Walter L Biffl

**Affiliations:** 1 From The Department of Surgery, Denver Health Medical Center, Denver CO, USA; 2 Surgical Intensive Care Unit, Trauma & Acute Care Surgery Fellowship, Department of Surgery, Denver Health Medical Center, 777 Bannock Street, MC 0206, Denver, CO 80204, USA

**Keywords:** Trauma, Injury, Spleen, Blush, Contrast extravasation, Angioembolization

## Abstract

**Background:**

Currently, evidence of contrast extravasation on computed tomography (CT) scan is regarded as an indication for intervention in splenic injuries. In our experience, patients transferred from other institutions for angioembolization have often resolved the blush upon repeat imaging at our hospital. We *hypothesized *that not all splenic blushes require intervention.

**Methods:**

During a 10-year period, we reviewed all patients transferred with blunt splenic injuries and contrast extravasation on initial postinjury CT scan.

**Results:**

During the study period, 241 patients were referred for splenic injuries, of whom 16 had a contrast blush on initial CT imaging (88% men, mean age 35 ± 5, mean ISS 26 ± 3). Eight (50%) patients were managed without angioembolization or operation. Comparing patients with and without intervention, there was a significant difference in admission heart rate (106 ± 9 *vs *83 ± 6) and decline in hematocrit following transfer (5.3 ± 2.0 *vs *1.0 ± 0.3), but not in injury grade (3.9 ± 0.2 *vs *3.5 ± 0.3), systolic blood pressure (125 ± 10 *vs *115 ± 6), or age (38.5 ± 8.2 *vs *30.9 ± 4.7). Of the 8 observed patients, 3 underwent repeat imaging immediately upon arrival with resolution of the blush. In the intervention group, 4 patients had ongoing extravasation on repeat imaging, 2 patients underwent empiric embolization, and 2 patients underwent splenectomy for physiologic indications.

**Conclusions:**

For blunt splenic trauma, evidence of contrast extravasation on initial CT imaging is not an absolute indication for intervention. A period of observation with repeat imaging could avoid costly, invasive interventions and their associated sequelae.

## Introduction

A contrast blush on computed tomography (CT) scan has been identified as a risk factor for failure of nonoperative management (NOM) of splenic injuries [1-3], prompting many centers to perform routine splenic artery angioembolization in the presence of a blush [4,5]. Using evidence of contrast extravasation on CT scan as an indication for angioembolization, however, has never been subjected to rigorous analysis. In our experience, patients with splenic injuries transferred from other institutions specifically for angioembolization have often resolved the blush upon repeat imaging at our hospital. This made us question whether all postinjury splenic blushes were equivalent. Is evidence of contrast blush a mandate for intervention, or are there some injuries that cease active bleeding due to "internal tamponade" within the substance of the spleen? And how does one differentiate such patients? We *hypothesized *that not all splenic blushes require intervention and that patients may be selectively observed based upon physiologic status.

## Materials and methods

During a 10 year period, all patients transferred from an outside hospital with blunt splenic injuries and evidence of active contrast extravasation on initial postinjury CT scan were evaluated. Patients undergoing intervention (angioembolization or splenectomy) were compared to those managed without intervention. Demographic data, laboratory values, vitals, intervention, and outcome were analyzed. Patients with identified pseudoaneurysms were excluded. Statistical analysis was performed using SAS for Windows (SAS Institute, Cory, NC); *p-value *< 0.05 was considered statistically significant. The Colorado Multi-Institutional Review Board approved this study.

## Results

During the study period, 241 patients with splenic injuries were transferred from an outside hospital, of which 16 had a contrast blush on CT imaging. All contrast blushes were intraparenchymal. The majority (88%) of patients were men with a mean age of 35 ± 5 and mean ISS of 26 ± 3. Mean time of transfer to Denver Health following injury and evaluation at an outside hospital was 6.4 ± 1.5 h. One patient received 1 unit of packed red blood cells during transfer. No patient reported use of anticoagulant or antiplatelet medications. Eight (50%) of these sixteen patients were managed without angioembolization or operation. In the group not undergoing intervention, Focused Abdominal Sonography for Trauma (FAST) examination was positive in six and negative in two patients. In patients undergoing intervention, FAST was positive in two patients and was not performed in the remainder. The two groups of patients had similar splenic AAST injury grades, age, injury severity scores, and emergency department systolic blood pressure (Table 1). The amount of intraperitoneal blood did not appear to be different between the two groups. The group managed without intervention had 1 patient with left upper quadrant (LUQ) blood, 5 patients with bilateral upper quadrant (BUQ) free fluid, and 2 patients with blood extending into the pelvis. In the group undergoing intervention, 3 patients had BUQ free fluid, and 3 patients had blood extending into the pelvis; the remaining 2 patients had no comment of intraperitoneal free fluid noted. In patients undergoing intervention there was a significant difference in admission heart rate and decline in hematocrit following transfer compared to patients who did not require operation or angioembolization (Table 1).

**Table 1:** Patient demographics and injury characteristics stratified by management technique

	Injury Grade	Age	ISS	SBP in the ED	HR in the ED	Decline in hematocrit following transfer
**Nonoperative Management** **(N = 8)**	3.5 ± 0.3	30.9 ± 4.7	26.8 ± 4.2	115 ± 6	83 ± 6	1.0 ± 0.3

**Intervention** **(N = 8)**	3.9 ± 0.2	38.5 ± 8.2	25.5 ± 4.6	125 ± 10	106 ± 9*	5.3 ± 2.0*

*ISS *Injury Severity Score, *SBP *systolic blood pressure, *HR *heart rate *p-value < 0.05

*ED *Emergency Department

**Figure 1:**
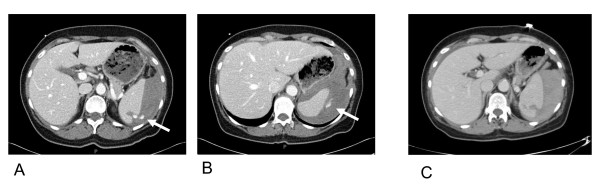
**CT scans from the outside hospital demonstrate contrast extravasation from the spleen (A,B)**. Repeat imaging at Denver Health reveals the blush has resolved (c).

In the 8 (50%) patients managed with observation, 3 underwent repeat imaging immediately after transfer; CT scan revealed the blush had resolved (Figure 1). None required blood product transfusion. Of these 8 patients there was 1 complication; a 49 year-old man with a grade III splenic laceration which had been stable without extravasation on repeat CT scan imaging had a delayed bleed on hospital day #4 treated with angioembolization. Eight (50%) patients underwent intervention following transfer (5 angioembolizations and 3 splenectomies). Two patients underwent immediate angiography without repeat CT scanning; although there was no evidence of contrast extravasation they underwent empiric main splenic artery embolization. Four patients had evidence of ongoing extravasation on repeat CT scan imaging and underwent intervention (3 angioembolization and 1 splenectomy). Two patients underwent immediate splenectomy upon arrival to DHMC based upon clinical indices. The eight patients received a mean of 3 ± 1.6 units of packed red cells during hospitalization. None of the eight patients had a splenic related complication. There were no significant differences in ventilator days, ICU length of stay, or hospital length of stay between the intervention and observation groups.

## Discussion

Angioembolization has been reported to increase the success rates of NOM of splenic injuries [5-10]. One scenario which is considered by some to be an absolute indication for angioembolization is the hemodynamically stable patient demonstrating a contrast blush on admission CT scan. It is a logical presumption that evidence of arterial bleeding, a contrast blush, seen on CT imaging would decrease the likelihood of spontaneous hemostasis. In fact, patients with a splenic injury and an associated contrast blush are reportedly 24 times more likely to fail NOM [1]. Further study by Federle et al. noted a 19% incidence of contrast blush in their patient population of which only 7% were successful in NOM [2]. Therefore, angiography for patients manifesting a blush associated with their splenic injury has been recommended [11]. However, these data do not answer the question of whether all patients with evidence of contrast extravasation from splenic injury mandate intervention. Angioembolization is invasive, costly, and complications occur in over 20% of patients [8,12-14].

In our experience, half of patients with a contrast blush on initial postinjury CT scan did not require intervention, either operative or catheter based, following transfer to our hospital for intended angioembolization. This number may, in fact, have been higher if the two patients who did not show evidence of extravasation at angiography but underwent empiric embolization were considered in this group rather than the treatment group. Those patients that underwent intervention had significantly higher ED heart rates and decline in their post-transfer hematocrit. Similar to our findings, Omert et al. reported that a patient's hemodynamics are more predictive of the need for intervention than contrast blush alone [15]. They describe the successful NOM of nine patients with splenic injuries and contrast blush, concluding that the mere presence of a contrast blush was not an absolute indication for intervention. Similar conclusions in children have also been reported [16]. Unlike other studies that have shown a correlation between increasing AAST splenic injury grade, increased incidence of contrast blush, and need for intervention [1], our group showed similar injury grades between those undergoing NOM and those requiring intervention.

There are inherent limitations in any retrospective evaluation. Additionally, the numbers in this series may be considered small, hence precluding broad generalization. However, this study serves to underscore that the surgical dictum, all blushes require embolization, may not be supported by scientific evidence once evaluated. This study is small due to the catchment population - only those patients with outside facility imaging demonstrating a blush associated with a splenic injury were included. We purposefully excluded those patients whose first evaluation was in our own emergency department with subsequent admission as management along the "surgical dictum" was more probable. By analyzing those patients who underwent transport times and hence permitted a repeated and delayed evaluation, gave us a time-frame without intervention. Finally, specifics of CT imaging technique at the outside hospital were not obtained, such as contrast volume, rate of infusion, slice thickness, and presence of delayed images; if the outside facility's physicians felt the imaging supported a diagnosis of a splenic blush mandating transport to a level I trauma center we felt specifics were of secondary importance.

## Conclusions

With increases in technology and high resolution CT imaging, it is likely that more contrast blushes will be detected. Assuming that a hemodynamically stable patient requires angiography for investigation of a contrast blush is not based on scientific evidence. Based upon our experience, albeit limited in numbers and retrospective in nature, we do not feel evidence of contrast extravasation on initial CT imaging alone is a definitive indication for intervention. A period of close observation, serial examination, repeat laboratory evaluation, repeat FAST for those with an initial negative FAST, and selective repeat CT imaging, should be considered. A clinically based approach, similar to that used in all patients to determine operative versus NOM of blunt splenic injuries, rather than immediate angiography could avoid costly, invasive interventions and their associated sequelae. Future prospective trials would help delineate patients with splenic blushes who can be managed non-operatively, and could help develop treatment algorithms.

## Competing interests

The authors declare that they have no competing interests.

## Authors' contributions

Study Design: B Data Collection/Analysis/Interpretation: B, K, M. Manuscript Drafting: B, K, M. Critical Review: B, J. All authors read and approved the final manuscript.
